# Pregnancy Deciduosis Mimicking Abdominal Tuberculosis and Peritoneal Carcinomatosis: A Case Report

**DOI:** 10.3390/diagnostics16183015

**Published:** 2026-09-17

**Authors:** Andrea Musarò, Maria Paola Bonasoni, Mariangela Pati, Emanuela Carossino, Alice Ferretti, Benedetta Petrachi, Alberto Cavazza, Alessia Papapietro, Ione Tamagnini, Marialisa Marchetti, Lorenzo Aguzzoli

**Affiliations:** 1 Unit of Obstetrics and Gynecologic Oncology, Azienda USL-IRCCS di Reggio Emilia, 42122 Reggio Emilia, Italy; andrea.musaro@ausl.re.it (A.M.); mariangela.pati@ausl.re.it (M.P.); emanuela.carossino@ausl.re.it (E.C.); alice.ferretti@ausl.re.it (A.F.); benedetta.petrachi@ausl.re.it (B.P.); lorenzo.aguzzoli@ausl.re.it (L.A.); 2 Pathology Unit, Azienda USL-IRCCS di Reggio Emilia, 42122 Reggio Emilia, Italy; alberto.cavazza@ausl.re.it (A.C.); alessia.papapietro@ausl.re.it (A.P.); ione.tamagnini@ausl.re.it (I.T.); marialisa.marchetti@ausl.re.it (M.M.)

**Keywords:** deciduosis, miliary tuberculosis, abdominal carcinomatosis, mesothelioma

## Abstract

**Background and clinical significance**: Gestational deciduosis is a benign, progesterone-related ectopic decidual reaction that commonly involves pelvic and peritoneal surfaces and usually regresses after delivery. Although generally asymptomatic, diffuse peritoneal deciduosis may present as multiple nodular implants and closely mimic peritoneal carcinomatosis, mesothelioma, or miliary tuberculosis, creating a challenging intraoperative differential diagnosis. Prompt histopathological assessment is essential to prevent unnecessary staging procedures or oncological treatment. **Case presentation**: A 30-year-old primigravida at 40 weeks’ gestation underwent unplanned intrapartum cesarean section after a premature rupture of membranes, the induction of labor, and failed progression. Multiple friable, gray–yellow subcentimetric nodules were incidentally observed on the uterine serosa, adnexa, omentum, visceral intestinal peritoneum, and appendix. The macroscopic appearance raised concern for peritoneal carcinomatosis and abdominal miliary tuberculosis. No ascites or intra-abdominal masses were identified. CA-125 was elevated (207.7 U/mL), Quantiferon testing was indeterminate, and postoperative chest computed tomography showed no pulmonary disease. Histological examination showed ectopic decidual cells with abundant eosinophilic cytoplasm in a perivascular and septal distribution. The cells showed diffuse vimentin, CD10, and progesterone-receptor expression and lacked epithelial and mesothelial marker expression, supporting diffuse peritoneal deciduosis. The postoperative course was uncomplicated, and pelvic ultrasonography at 40 days postpartum showed no residual lesions. **Conclusions**: Diffuse peritoneal deciduosis should be considered when disseminated peritoneal nodules are encountered during pregnancy or cesarean delivery. In this setting, elevated CA-125 is nonspecific and may heighten concern for tuberculosis or malignancy without distinguishing among these entities. Correlation of the clinical context, operative findings, morphology, and targeted immunohistochemistry is essential to avoid unnecessary oncological treatment or extensive staging procedures.

## 1. Introduction

Ectopic decidual reaction, also known as deciduosis, is a benign, most often pregnancy-associated condition characterized by ectopic decidual tissue on peritoneal surfaces of abdominal and pelvic organs, resulting from progesterone-induced metaplasia of subperitoneal mesenchymal cell [1,2]. Deciduosis is classically described in the pelvis and genital tract, such as the uterine serosa, fallopian tubes, ovaries, cervix, and vagina. However, it has also been documented at multiple extra-pelvic sites including peritoneum [3], omentum [4], appendix [5,6], lymph nodes [7], lungs [8], kidneys [9], and even skin [10].

Although microscopic ectopic decidual change may be encountered incidentally during pregnancy, grossly apparent diffuse peritoneal deciduosis is uncommon and has been documented predominantly in individual case reports and small series. This physiologic phenomenon has been found in approximately 10% of cesarean sections (CS) [2], but in most women, the condition remains clinically silent and typically regresses spontaneously 4–6 weeks postpartum [1,4]. Its macroscopic appearance may be striking and, when florid, can closely simulate malignant processes such as peritoneal carcinomatosis or malignant mesothelioma, creating a major intraoperative diagnostic dilemma [11,12].

Herein, we report a case of diffuse peritoneal deciduosis in a full-term primigravida incidentally discovered during an unplanned intrapartum CS, mimicking abdominal miliary tuberculosis, raising the suspicion of carcinomatosis, and posing a significant intraoperative diagnostic challenge.

## 2. Case Presentation

A 30-year-old primigravida presented at 40 weeks of gestation with premature rupture of membranes. Her medical history was notable for chronic thyroiditis and multiple known uterine fibroids, with the largest located anteriorly and measuring 56 × 48 × 45 mm. She had no other significant comorbidities and no prior surgical history. During the first trimester, she required hospitalization for hyperemesis gravidarum. Subsequently, she developed hypothyroidism, for which she was started on replacement therapy. First-trimester screening with cell-free fetal DNA testing indicated a high risk for trisomy 13; however, confirmatory amniocentesis demonstrated a normal fetal karyotype (46, XY). At term, laboratory investigations revealed elevated liver transaminases (ALT 119 U/L, AST 47 U/L), which normalized spontaneously on repeat testing.

At admission, the amniotic fluid was clear, and the patient was afebrile. A vagino-rectal swab tested negative for Group B beta-hemolytic Streptococcus. Expectant management was therefore adopted for 24 h, with antibiotic prophylaxis initiated 18 h after membrane rupture. After 24 h, in the presence of a favorable obstetric examination and following informed consent, labor was induced with intravenous oxytocin.

Intraoperatively, multiple subcentimeter grayish-yellow nodular implants were incidentally identified. These lesions were friable and prone to bleeding on contact, involving the uterine serosa, adnexa, omentum, intestinal visceral peritoneum, and appendix. Multiple biopsies were obtained and sent for histopathological evaluation. Estimated intraoperative blood loss was approximately 600 mL. A healthy male neonate was delivered, weighing 3242 gr, with Apgar scores of 9 and 10 at 1 and 5 min, respectively. Initially, the intraoperative findings raised a suspicion of peritoneal carcinomatosis; however, both adnexa were grossly unremarkable with no evident ovarian masses, tubal lesions, ascites, or other intra-abdominal masses. Moreover, the patient had no personal history of malignancy. Her family history was negative for breast, ovarian, endometrial, colorectal, pancreatic cancer or other hereditary cancer syndromes. No previous genetic counseling or germline testing had been indicated. Tumor marker evaluation was unremarkable except for an elevated CA-125 level (207.7 U/mL; reference value <30 U/mL). As this marker can also be elevated in miliary tuberculosis [13], other than carcinomatosis, the former was considered as a differential diagnosis. The patient had no history of tuberculosis, known exposure to active tuberculosis, immunosuppressive treatment, or constitutional or respiratory symptoms suggestive of active disease. However, her epidemiological background—African ancestry and residence in, with multiple trips to, a sub-Saharan African country—prompted consideration of tuberculosis in the intraoperative differential diagnosis. A Quantiferon test yielded an indeterminate result, and a chest computed tomography (CT) scan performed in the immediate postoperative period showed no evidence of pulmonary involvement. Although an abdominal CT scan was recommended, the patient declined; therefore, an abdominal ultrasound was performed, confirming appendiceal involvement.

The postoperative course was uneventful, and the patient was discharged in good clinical conditions on postoperative day three. At the 40-day postpartum follow-up, the patient remained in good general health, with normal postoperative findings and no evidence of residual lesions on pelvic ultrasound.

**Figure 1:**
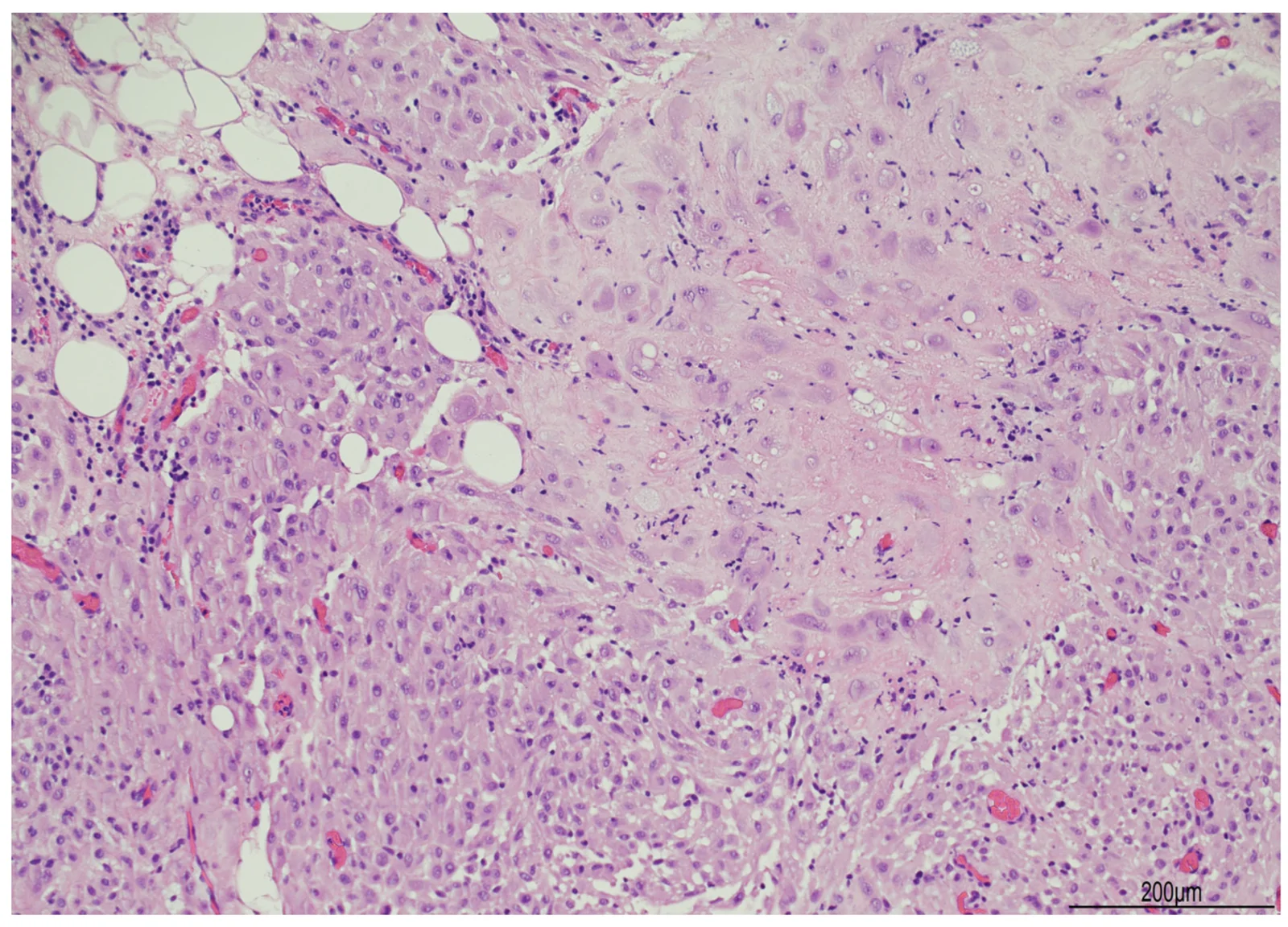
Peritoneal deciduosis: Surgical biopsies showed large, polyhedral cells with eosinophilic cytoplasm, and round nuclei with prominent nucleoli. These cells mimicked peritoneal fat infiltration (hematoxylin and eosin, 10 HPF).

A histopathological analysis of the biopsied lesions displayed benign ectopic decidua appearing as large, polyhedral cells with eosinophilic cytoplasm, and round nuclei with prominent nucleoli. These cells clustered along the interlobular septa of the adipose tissue, mimicking peritoneal fat infiltration. Vascular compartment with capillaries and small arterioles was also prominent (Figure 1).

Immunohistochemistry was carried out with the Roche-Ventana automated system. The following antibodies were tested: pancytokeratin (CKAE1/AE3), cytokeratin 5 (CK5), vimentin, S100, calretinin, CD68PGM1, HMB45, WT1, CD10, desmin, smooth muscle actin (SMA), estrogen receptor (ER), progesterone receptor (PR), D2-40 (podoplanin), and PAX8. All the relevant features, clones and manufacturers are listed in Table 1.

**Figure 2:**
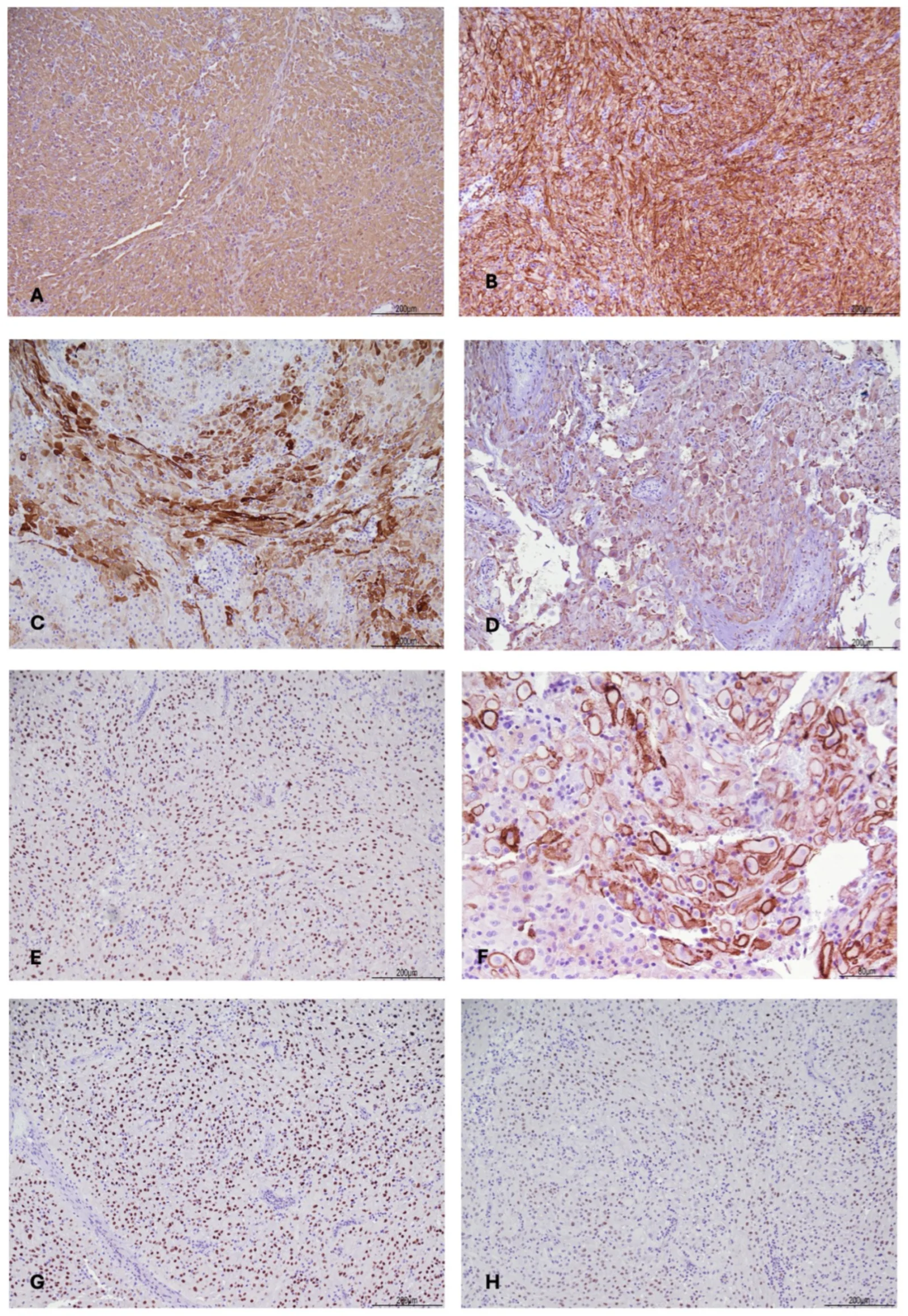
Immunohistochemical results of deciduosis panel: The decidual cells were diffusely positive for vimentin (**A**), CD10 (**B**), focally positive for desmin (**C**) and CD68 PGM1 (**D**); the progesterone receptor was diffusely positive (**E**); D2-40 (podoplanin) was focal (**F**); WT-1 was overall expressed (**G**), and the estrogen receptor was spottily positive. Magnifications: (**A**–**E**,**G**,**H**) 10 HPF; (**F**) 20 HPF.

Immunohistochemical staining showed the following patterns of positive expression: vimentin (strong positivity, diffuse, cytoplasmic); WT1 (positive, nuclear), CD68PGM1 (positive, scattered, cytoplasmic), CD10 (strong positivity, diffuse, membranous), desmin (positive, scattered, cytoplasmic), ER (focal, scattered, nuclear), PR (strong positivity, diffuse, nuclear), D240 (focal positivity, membranous) (Figure 2). The other antibodies turned out to be negative (CKAE1/AE3, CK5, calretinin, S100, SMA, HMB45, PAX8, BerEP4).

## 3. Discussion

Gestational deciduosis is a well-recognized benign condition defined by the presence of ectopic decidual tissue outside the uterine cavity during pregnancy. First described in 1864 by Walker [14], it is most commonly identified incidentally during CS [2], although some authors consider it a near-universal physiological phenomenon of pregnancy [4]. Several pathogenetic mechanisms have been proposed. The most widely accepted hypothesis, advanced by Zaytsev and Taxy [1], suggests that elevated progesterone levels during pregnancy induce a metaplastic transformation of pluripotent subcoelomic mesenchymal cells into decidual cells. This mechanism is supported by both morphological and clinical observations [2,12].

Gestational deciduosis shares certain features with endometriosis, including the hormonal response as well as the potential involvement of extra-abdominal sites [8]. However, unlike endometriosis, deciduosis does not result in architectural distortion of tissues and demonstrates spontaneous regression following delivery. In our case, the patient showed elevated CA-125 levels without any clinical history or symptoms suggestive of endometriosis. To the best of our knowledge, this may represent the first reported case of deciduosis associated with CA-125 elevation. CA-125 is a nonspecific biomarker that may be elevated in physiological pregnancy and in a wide range of benign and malignant conditions involving Müllerian or mesothelial-lined surfaces. Increased concentrations have been described in endometriosis, pelvic inflammatory conditions, ascites, peritoneal tuberculosis, and ovarian or primary peritoneal malignancies [15,16,17]. Consequently, the CA-125 value of 207.7 U/mL in the present case could not discriminate between deciduosis, peritoneal tuberculosis, and carcinomatosis. Although the precise mechanism cannot be determined, pregnancy-related mesothelial activation and extensive peritoneal involvement may have contributed.

Clinically, gestational deciduosis is often asymptomatic and discovered incidentally. Nevertheless, symptomatic presentations have been described, including acute abdomen due to appendiceal involvement [5,6,18], bowel obstruction [19] or hemoperitoneum [20], as well as chronic mild-to-moderate abdominal pain. In our patient, severe hyperemesis gravidarum requiring hospitalization occurred during the first trimester. Hyperemesis gravidarum and deciduosis may both occur in the context of pregnancy-related hormonal changes, but our case cannot provide a certain and causal association between hyperemesis, progesterone concentration, and diffuse peritoneal deciduosis.

Two forms of gestational deciduosis are recognized: localized and diffuse [3,21]. The localized form is far more common, whereas the diffuse form, as observed in our case, is rare and typically associated with heightened hormonal stimulation, such as in multiple pregnancies [3] or exogenous progestin exposure. The diffuse form is considerably less frequently reported than localized decidual reactions. The published evidence is dominated by case reports and small series, which precludes a reliable incidence estimate and supports the clinical relevance of reporting carefully documented cases with extensive peritoneal involvement. A reasonable numeric frequency to quote for intraoperatively encountered (macroscopic) deciduosis at cesarean is ~10% (31/307) in a prospective consecutive CS series [2].

Rare cases have also been reported outside pregnancy, as observed in patients with autoimmune disorders [11]. It may also be potentially observed in other clinical settings, such as progesterone-secreting adrenal tumors or during hormone replacement therapy. From a macroscopic standpoint, diffuse deciduosis can pose a significant diagnostic challenge. Peritoneal carcinomatosis was considered because the disseminated peritoneal nodules had an alarming gross appearance [12]. This possibility was prompted by the intraoperative morphology rather than by a known malignancy or a specific hereditary cancer risk. The absence of ascites, a dominant abdominopelvic mass, and grossly suspicious adnexal lesions reduced the clinical likelihood of peritoneal malignancy, but definitive exclusion required histopathological examination.

Instead, abdominal tuberculosis was initially considered intraoperatively because diffuse, small, gray–yellow peritoneal nodules may macroscopically resemble tuberculous implants. This differential diagnosis was driven by an operative appearance, rather than a preoperative clinical diagnosis or a positive microbiological/immunological test. The indeterminate interferon-gamma release assay (Quantiferon) was non-diagnostic, and the final diagnosis was established histologically and immunohistochemically.

Histologically, deciduosis is characterized by large polygonal cells with abundant eosinophilic cytoplasm and varying degrees of vacuolar change [3,22]. Immunohistochemistry is essential for differential diagnosis. Typically, deciduosis demonstrates positivity for CD10 and CD68, and negativity for smooth muscle actin (SMA) and cytokeratins (CK5/6, CK AE1/AE3), helping to distinguish it from malignancies such as carcinoma or mesothelioma [11,22]. In our case, immunohistochemistry revealed the positive expressions of vimentin (diffuse, strong cytoplasmic positivity), WT1 (nuclear positivity), CD68/PGM1 (scattered cytoplasmic positivity), CD10 (scattered cytoplasmic positivity), desmin (scattered cytoplasmic positivity), ER (focal, scattered nuclear positivity), and PR (diffuse, strong nuclear positivity) (Figure 2). All other markers tested—CK AE1/AE3, CK5, calretinin, S100, SMA, and HMB45—were negative.

Because decidual cells can show plump, vacuolated, or mildly atypical morphology, deciduosis is a recognized histologic mimic of both malignant mesothelioma (including its deciduoid variant) and ovarian/peritoneal serous carcinoma [23,24,25,26]. Misdiagnosis has serious consequences—overcalling malignancy can lead to unnecessary staging surgery or chemotherapy in a pregnant patient, while undercalling a true malignancy delays treatment.

Immunohistochemistry panels help differentiate the two neoplastic entities from deciduosis other than morphology. The main immunohistochemical markers are thoroughly illustrated in Table 2.

Calretinin and CK5/6 are the gold standard of the mesothelial immunopanel. Calretinin, which shows both nuclear and cytoplasmic (granular) staining, offers the strongest overall diagnostic profile: pooled sensitivity of 91% and specificity of 96% for malignant mesothelioma in serous effusion specimens [27], confirmed by immunohistochemical markers in malignant pleural mesothelioma and epithelioid mesothelioma [28], with cross-reactivity against ovarian serous carcinoma remaining low, in the range of 8–10% [29]. CK5/6, a cytoplasmic stain, by contrast, is highly sensitive for epithelioid mesothelioma (51–100%) but far less reliable in the sarcomatoid subtype, where sensitivity drops sharply to 13–29% [30,31]. It shows the greatest cross-reactivity with serous carcinoma, with reported positivity ranging from 25% [29] to 55% [24] a limitation that undermines its standalone specificity.

**Table 2:** Literature-informed immunohistochemical features useful in the differential diagnosis of peritoneal deciduosis, malignant mesothelioma, and serous carcinoma.

Marker	Pattern	Our Case of Deciduosis	Deciduosis in Literature	Malignant Mesothelioma/Serous Carcinoma
**D2-40 (Podoplanin)**	Membranous	Focal, membranous positivity	Intensely, diffusely positive in decidualized stromal cells [30].	Highly sensitive for epithelioid mesothelioma (80–100%) vs. lower positivity in serous carcinoma (15–65%) [20,23].
**Calretinin**	Nuclear and cytoplasmic (granular)	Negative	Negative, unlike the strongly positive native/hyperplastic mesothelium overlying the lesion [22].	Positive in mesothelioma (sensitivity ≈91%, specificity ≈96%); low cross-reactivity in serous carcinoma (≈8–10%)—most useful of these markers [28,29].
**CK5/6**	Cytoplasmic	Negative (CK5 tested)	Typically negative; rare focal positivity reported—do not use in isolation [26].	Sensitive for epithelioid mesothelioma (51–100%), far less in sarcomatoid (13–29%); greatest cross-reactivity with serous carcinoma (25–55%) [24,29,30,31].
**Pankeratin (AE1/AE3)**	Cytoplasmic	Negative	Negative to weak/focal; a subset can show unexpected focal positivity [22,26].	Diffusely positive in both entities [26].
**Vimentin**	Cytoplasmic	Strong, diffuse positivity	Diffusely positive [4,11,12,19].	Usually negative to weak in mesothelioma/serous carcinoma [22].
**Progesterone receptor (PR)**	Nuclear	Strong, diffuse positivity	Strongly, diffusely positive [3,11,12,19].	Negative in mesothelioma; positive in ~95% of serous carcinoma—limited standalone value [32].
**CD10**	Membranous/cytoplasmic	Strong, diffuse positivity	Positive [6,12].	Not a mesothelial/serous marker; supportive only [11,22].
**PAX8**	Nuclear	Negative	Negative	Negative in mesothelioma (0% pleural, ~9% peritoneal); positive in ≈99–100% of serous carcinoma—key tie-breaker [23,29].
**Ber-EP4**	Membranous	Negative	Negative	Negative in mesothelioma; positive in serous carcinoma (Ber-EP4 ≈95% sensitivity, 91% specificity) [32,33,34].
**WT1**	Nuclear	Positive	Negative in decidual cells (used to help exclude mesothelioma); may highlight adjacent native mesothelium [11,35].	Positive in most mesotheliomas and serous carcinomas; never use alone—pair with PAX8 or Ber-EP4/MOC-31 [32].

In the specific context of decidual mimickers, both markers are typically negative in decidual cells, in clear contrast to the strongly calretinin- and CK5/6-positive native or hyperplastic mesothelium that often overlies the lesion [11,35]. However, deciduoid mesothelioma can show CK5/6 positivity [26].

D2-40 (podoplanin), a membranous stain, shows high sensitivity for epithelioid mesothelioma, displaying positivity in 80–100% of cases, whereas its positivity in serous carcinoma is considerably lower, ranging from 15 to 65%. This differential was illustrated well in a direct comparative series, where the marker stained positive in 93% of mesotheliomas, compared with only 13% of serous carcinomas, underscoring its value in distinguishing between the two entities [23,26].

D2-40-positive expression has been described in normal decidual cells [36] but not validated in deciduosis.

Pankeratin (CKAE1/AE3), a typical cytoplasmic expression, is positive in both mesothelioma and serous carcinoma [26] but negative [22] or focally positive [11] in deciduosis.

Vimentin, expressed in the cytoplasm, is usually negative to weak in mesothelioma/serous carcinoma [25] but diffusely positive in deciduosis [4,11,12,22].

PR, nuclear positivity, is reported with high expression in serous carcinoma and deciduosis [3,11,12,22] but negative in mesothelioma [32].

CD10, membranous/cytoplasmic, is positive in deciduosis [6,12] but negative in serous carcinoma and mesothelioma.

PAX8, which shows nuclear expression, is the gold standard for distinguishing serous carcinoma (99% positivity) from mesothelioma (0–9% positivity in the peritoneal entity) [23,29] but usually negative in non-Müllerian, non-mesothelial lesions like deciduosis [29]), although a weak positivity has been reported [8].

BerEP4, a membranous stain, is negative in mesothelioma, even in the deciduoid variant, but positive in serous carcinoma (Ber-EP4 ≈ 95% sensitivity, 91% specificity) [26,33,34].

WT1 nuclear positivity is generally considered a mesothelial/serous-carcinoma marker feature [37,38] but is reported negative in deciduosis [11,35].

The immunohistochemical pattern of our case reflected what was reported in the literature for deciduosis: diffuse positivity for vimentin, PR, and CD10 [3,4,6,11,12,18]. Instead, negativity for PAX8, Ber-EP4, and calretinin was inconsistent with either mesothelioma or serous carcinoma [19,20,22,23,24,25,31,35]. Moreover, CK5/6 and CKAE1/AE3 negativity strengthened the diagnosis of deciduosis [18].

Notably, our case showed WT1 nuclear positivity, which contrasts with the negative WT1 expression generally reported in deciduosis [11,28]. D2-40 displayed focal membranous positivity, although this marker has never been included in the immunohistochemical panel for deciduosis.

However, both markers were described in the human decidual cells [36,39], especially WT-1 expression, which seems to indicate the early process of decidual differentiation [39].

In our case, WT-1 and D2-40 expression may either suggest an early decidual differentiation or a mesothelial-like cellular lineage.

**Figure 3:**
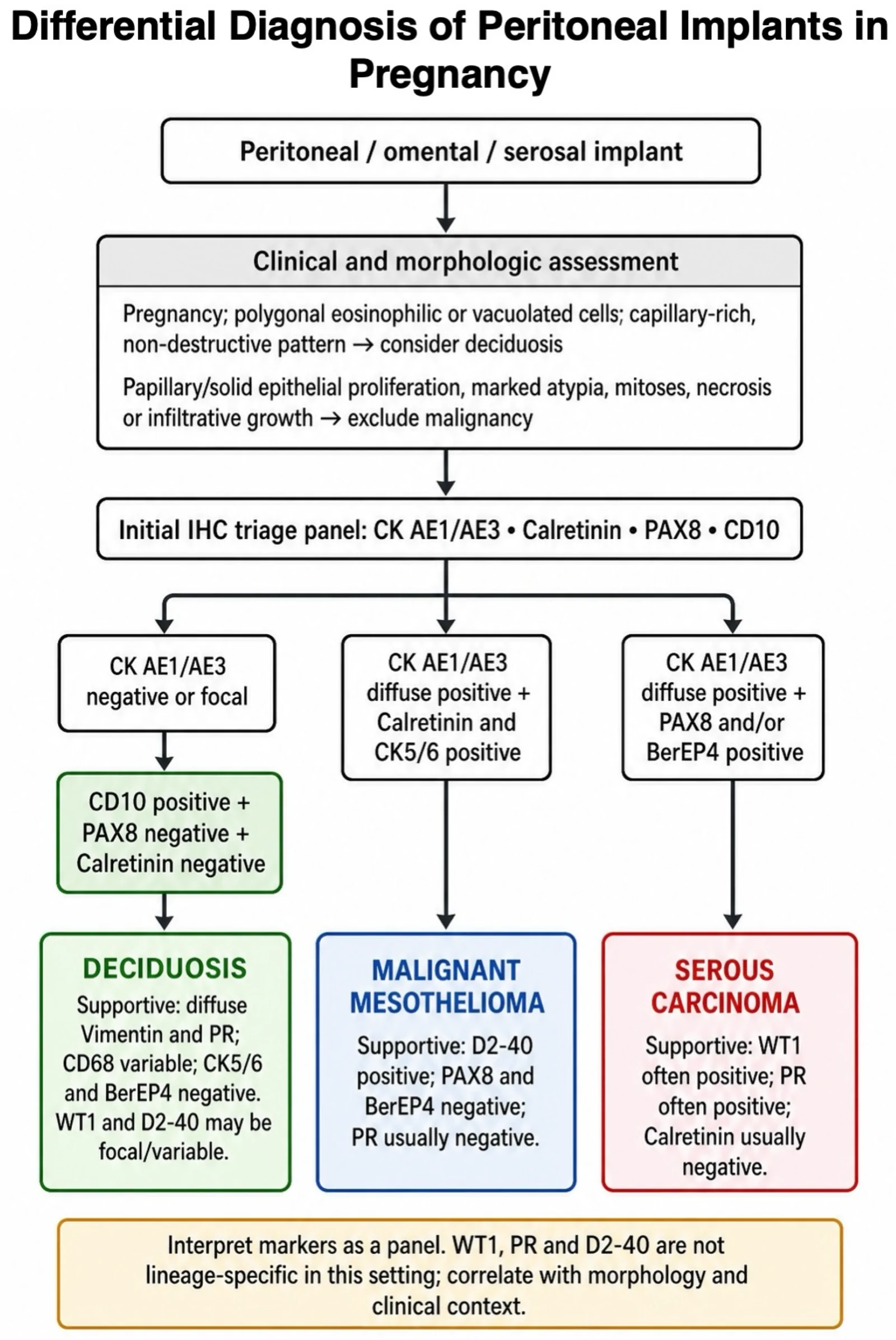
Practical literature-informed approach to the differential diagnosis of diffuse peritoneal deciduosis, malignant mesothelioma, and serous carcinoma.

Figure 3 provides a simplified, literature-informed schematic of the morphological and immunohistochemical considerations that may assist in distinguishing deciduosis from its principal malignant mimics. This figure is intended solely as an educational summary and has not been prospectively developed or independently validated as a diagnostic algorithm.

Gestational deciduosis is a self-limiting condition that resolves spontaneously within 4–6 weeks postpartum, following the decline in progesterone levels, and does not require specific treatment. In our case, postpartum follow-up confirmed complete resolution of the lesions, consistent with the natural history of the condition. However, recurrence may occur in the setting of renewed progesterone exposure. For this reason, targeted contraceptive counseling is recommended, favoring non-hormonal methods such as barrier contraception or a copper intrauterine device, while avoiding progestin-containing methods that could potentially exacerbate the condition.

Although benign, gestational deciduosis may recur in subsequent pregnancies. Prior recognition of the condition is therefore essential to ensure appropriate patient counseling and to avoid unnecessary diagnostic or therapeutic interventions.

## 4. Conclusions

Diffuse gestational deciduosis is a rare but benign condition that may closely mimic serious pathologies such as miliary tuberculosis or peritoneal carcinomatosis. Awareness of this entity and its distinguishing clinical, intraoperative, and histopathological features is crucial to establish an accurate diagnosis and to guide appropriate management.

## Figures and Tables

**Table 1:** Primary antibodies used, clones, and manufacturers.

Antibody	Clone	Company
Estrogen Receptor (ER)	SP1 Rabbit Monoclonal Primary Antibody	Ventana Medical Systems, Inc. 1910 E. Innovation Park Drive, Tucson, Arizona 85755, USA
Progesterone Receptor (PR)	1E2 Rabbit Monoclonal Primary Antibody	Ventana Medical Systems, Inc. 1910 E. Innovation Park Drive, Tucson, Arizona 85755, USA
Pan Keratin (CK PAN)	AE1/AE3/PCK26 cocktail of three monoclonal antibody clones	Ventana Medical Systems, Inc. 1910 E. Innovation Park Drive, Tucson, Arizona 85755, USA
Cytokeratin 5 (CK5)	SP27 Rabbit Monoclonal Primary Antibody	Cell Marque Corporation, 6600 Sierra College Blvd., Rocklin, CA 95677, USA
Desmin	DE-R-11 Mouse Monoclonal Primary Antibody	Ventana Medical Systems, Inc. 1910 E. Innovation Park Drive, Tucson, Arizona 85755, USA
Calretinin	SP65 Rabbit Monoclonal Primary Antibody	Ventana Medical Systems, Inc. 1910 E. Innovation Park Drive, Tucson, Arizona 85755, USA
CD10	SP67 Rabbit Monoclonal Primary Antibody	Ventana Medical Systems, Inc. 1910 E. Innovation Park Drive, Tucson, Arizona 85755, USA
S100	4C4.9 Mouse Monoclonal Primary Antibody	Ventana Medical Systems, Inc. 1910 E. Innovation Park Drive, Tucson, Arizona 85755, USA
WT1	6F-H2 Mouse Monoclonal Primary Antibody	Cell Marque Corporation, 6600 Sierra College Blvd., Rocklin, CA 95677, USA
Smooth Muscle Actin (SMA)	1A4 Mouse Monoclonal Primary Antibody	Cell Marque Corporation, 6600 Sierra College Blvd., Rocklin, CA 95677, USA
Vimentin	V9 Mouse Monoclonal Primary Antibody	Ventana Medical Systems, Inc. 1910 E. Innovation Park Drive, Tucson, Arizona 85755, USA
Melanosome (HMB45)	HMB45 Mouse Monoclonal Primary Antibody	Ventana Medical Systems, Inc. 1910 E. Innovation Park Drive, Tucson, Arizona 85755, USA
CD68 Macrophage	PG-M1 Mouse Monoclonal Primary Antibody	DBS, Pleasanton, CA, USA/MedEnvoy Global B.V.
D2-40 Podoplanin	D2-40 Mouse Monoclonal Primary Antibody	Cell Marque Corporation, 6600 Sierra College Blvd., Rocklin, CA 95677, USA
PAX8	EP331 Rabbit Monoclonal Primary Antibody	Cell Marque Corporation, 6600 Sierra College Blvd., Rocklin, CA 95677, USA
BerEP4 (Ep-CAM/Epithelial Specific Antigen)	Ber-EP4 Mouse Monoclonal Primary Antibody	Cell Marque Corporation, 6600 Sierra College Blvd., Rocklin, CA 95677, USA

## Data Availability

The data presented in this study are available upon request from the corresponding author.
